# Supplementary material for: High-Pressure Processing of Traditional Hardaliye Drink: Effect on Quality and Shelf-Life Extension
Source: Foods. 2023 Jul 28;12(15):2876. doi: 10.3390/foods12152876 (PMC10417461; doi:10.3390/foods12152876)
Supplement: Supplementary file 1 [file foods-12-02876-s001.zip › foods-2477196-supplementary.pdf]

Table S1. Changes in the physical properties of hardaliye during shelf-life studies.

		Storage Temperature			
		4°C		22°C	
	Days	Control	HHP treated	Control	HHP treated
pH	0	3.69±0.0^{Ba}	3.83±0.0^{Aa}	3.78±0.0^{A}	3.77±0.0^{Aa}
	15	3.52±0.0^{Bb}	3.85±0.0^{Aa}		3.76±0.0^{Ab}
	30		3.72±0.0^{Abc}		3.71±0.0^{Ab}
	45		3.74±0.0^{Ab}		3.75±0.0^{Aab}
	66		3.80±0.0^{Aab}		3.74±0.0^{Aab}
	87		3.66±0.1^{Ac}		3.72±0.1^{Ab}
	108		3.69±0.0^{Abc}		3.66±0.1^{Ab}
	142		3.68±0.1^{Abc}		
	180		3.68±0.0^{Abc}		
	228		3.69±0.0^{Abc}		
	Days	Control	HHP treated	Control	HHP treated
Conductivity (mS/cm)	0	3.42±0.0^{Aa}	3.42±0.0^{Aa}	3.43±0.03^{A}	3.42±0.0^{Aa}
	15	3.41±0.0^{Ba}	3.41±0.0^{Ba}		3.48±0.1^{Aa}
	30		3.50±0.0^{Aa}		3.50±0.0^{Aa}
	45		3.47±0.0^{Aa}		3.52±0.0^{Aa}
	66		3.51±0.0^{Aa}		3.52±0.0^{Aa}
	87		3.44±0.0^{Aa}		3.48±0.0^{Aa}
	108		3.45±0.0^{Ba}		3.51±0.0^{Aa}
	142		3.42±0.0^{Aa}		
	180		3.42±0.0^{Aa}		
	228		3.47±0.0^{Aa}		
	Days	Control	HHP treated	Control	HHP treated
Color *L**	0	11.81±1.6^{Aa}	11.59±0.9^{Aa}	9.48±0.8^{B}	11.81±1.6^{Aa}
	15	2.98±0.0^{Bb}	3.98±0.1^{Ab}		3.60±0.4^{Ab}
	30		3.79±0.4^{Ab}		3.67±0.5^{Ab}
	45		3.68±0.3^{Ab}		3.40±0.4^{Ab}
	66		3.52±0.2^{Ab}		3.33±0.4^{Ab}
	87		3.52±0.4^{Ab}		3.10±0.6^{Ab}
	108		3.53±0.3^{Ab}		3.13±0.5^{Ab}
	142		3.42±0.3^{Ab}		
	180		3.42±0.3^{Ab}		
	228		3.47±0.3^{Ab}		
	Days	Control	HHP treated	Control	HHP treated
Color *a**	0	32.96±0.6^{Aa}	32.75±3.2^{Aa}	32.84±0.9^{A}	32.82±1.9^{Aa}
	15	14.04±0.1^{Bb}	13.46±2.2^{Ab}		12.49±1.1^{b}
	30		12.68±1.2^{Ab}		8.78±1.1^{Ac}
	45		12.42±1.2^{Ab}		8.51±0.6^{Ac}
	66		12.49±1.2^{Ab}		5.95±0.2^{Ad}
	87		12.14±1.1^{Ab}		5.10±0.6^{Ad}
	108		12.16±1.3^{Ab}		5.11±0.5^{Ad}
	142		11.42±1.3^{Ab}		
	180		9.76±0.3^{Ac}		
	228		8.47±0.3^{Ad}		
	Days	Control	HHP treated	Control	HHP treated
Color *b**	0	12.87±1.6^{Aa}	12.19±1.4^{Aa}	12.26±1.0^{A}	13.80±2.6^{Aa}
	15	3.26±0.1^{Ab}	3.27±0.2^{Ab}		3.10±0.1^{Ab}
	30		3.25±0.1^{Ab}		2.49±0.1^{Bc}
	45		3.27±0.2^{Ab}		2.44±0.2^{Bc}
	66		3.24±0.1^{Ab}		2.47±0.3^{Bc}
	87		3.12±0.4^{Ab}		2.31±0.3^{Bc}
	108		3.09±0.7^{Ab}		2.36±0.2^{Bc}
	142		2.57±0.6^{Ac}		
	180		2.08±0.3^{Ac}		
	228		3.03±0.1^{Ac}		
	Days	Control	HHP treated	Control	HHP treated
	0	15.47±2.8^{Aa}	17.24±1.4^{Aa}	15.41±1.4^{A}	13.89±2.6^{Aa}

Chroma	15	12.41±0.41^{Ab}	13.81±0.5^{Ab}		13.86±0.4^{Aa}
	30		13.04±0.1^{Ab}		9.07±0.8^{Bb}
	45		12.85±0.2 ^{Abc}		6.87±0.6^{Bc}
	66		12.86±0.1 ^{Abc}		6.57±0.5 ^{Bc}
	87		12.52±0.1^{Abc}		6.50±0.4^{Bc}
	108		12.16±1.5^{Abc}		5.62±0.9^{Bd}
	142		11.99±1.3^{Abc}		
	180		10.36±0.8^{Abc}		
	228		9.98±0.2^{Ad}		
	Days	Control	HHP treated	Control	HHP treated
Hue	0	0.36±0.03^{Aa}	0.34±0.08^{Aa}	0.33±0.22^{A}	0.38±0.03^{Aa}
	15	0.23±0.01^{Ab}	0.22±0.01^{Ab}		0.24±0.01^{Ab}
	30		0.22±0.01^{Ab}		0.25±0.01^{Bb}
	45		0.23±0.03 ^{Ab}		0.23±0.03^{Ab}
	66		0.25±0.02 ^{Ab}		0.23±0.03^{Ab}
	87		0.24±0.02^{Ab}		0.22±0.02^{Ab}
	108		0.25±0.03^{Ab}		0.17±0.02^{Bd}
	142		0.24±0.03^{Ab}		
	180		0.23±0.02^{Ab}		
	228		0.21±0.04^{Ab}		
	Days	Control	HHP treated	Control	HHP treated
Total color difference	0	0.00±0.0^{Ba}	1.32±0.1^{Aa}	0.00±0.0^{B}	1.38±0.1^{Aa}
	15	0.21±0.0^{Ab}	1.22±0.1^{Aa}		1.24±0.1^{Aa}
	30		1.22±0.1^{Aa}		1.25±0.1^{Aa}
	45		1.20±0.1^{Aa}		1.20±0.1^{Aa}
	66		1.25±0.1^{Aa}		1.21±0.1^{Aa}
	87		1.24±0.2^{Aa}		1.22±0.1^{Aa}
	108		1.25±0.1^{Aa}		1.24±0.1^{Aa}
	142		1.24±0.1^{Aa}		
	180		1.23±0.1^{Aa}		
	228		1.21±0.1^{Aa}		
	Days	Control	HHP treated	Control	HHP treated
Color intensity	0	4.90±0.4^{Aa}	4.90±0.4^{Aa}	4.59±0.3^{A}	4.29±0.4^{Aa}
	15	3.87±0.^{Ab}	3.97±0.1^{Ab}		4.05±0.1^{Aa}
	30		4.16±0.2^{Ab}		4.39±0.2^{a}
	45		4.07±0.1 ^{Ab}		4.11±0.2^{Aa}
	66		4.00±0.2 ^{Ab}		4.29±0.2^{Aa}
	87		2.65±0.3^{Ac}		3.20±0.1^{Ab}
	108		2.28±0.2^{Ac}		3.24±0.2^{Ab}
	142		2.24±0.1^{Ac}		
	180		2.23±0.1^{Ac}		
	228		2.22±0.1^{Ac}		

Data in the same column with different lowercase superscript letter and data in the same row with uppercase superscript letter are significantly different ($p \leq 0.05$).

Table S2. Changes in the sensory properties of hardaliye during shelf-life studies.

		Storage Temperature			
		4°C		22°C	
	Days	Control	HHP treated	Control	HHP treated
Clarity-Cloudiness	0	9.33±0.9^{Aa}	9.60±0.4^{Aa}	9.00±0.5^{A}	9.88±0.43^{Aa}
	15	6.77±0.2^{Bb}	9.00±0.7^{Aa}		8.44±0.72^{Aa}
	30		8.60±0.8^{Aa}		8.33±0.20^{Aa}
	45		7.80±0.1 ^{Aab}		7.04±0.26^{Ab}
	66		7.68±0.3^{Ab}		6.32±0.14^{Bc}
	87		7.22±0.2^{Ab}		6.05±0.22^{Bc}
	108		6.98±0.3^{Ac}		5.94±0.20^{Bc}
	142		6.88±0.3^{Ac}		
	180		6.58±0.3^{Ac}		
	228		6.56±0.2^{Ac}		
	Days	Control	HHP treated	Control	HHP treated
Shininess-Dullness	0	9.22±0.38^{Aa}	9.66±0.41^{Aa}	9.21±0.5^{A}	9.33±0.3^{Aa}
	15	5.40±0.23^{Cb}	9.10±0.60^{Aa}		8.12±0.6^{Bab}
	30		8.96±0.46^{Aa}		8.00±0.2^{Ab}
	45		8.14±0.13^{Aab}		7.65±0.3^{Bb}
	66		8.08±0.30^{b}		7.50±0.2^{Bb}
	87		7.69±0.26^{Ab}		7.42±0.3^{Ab}
	108		7.62±0.24^{Ac}		7.02±0.3^{Ab}
	142		7.22±0.39^{Ac}		
	180		7.11±0.42^{Ac}		
	228		7.05±0.28^{Ac}		
	Days	Control	HHP treated	Control	HHP treated
Color intensity	0	7.33±1.6^{Aa}	8.46±0.4^{Aa}	7.33±0.4^{A}	8.22±0.4^{Aa}
	15	6.44±0.5^{Cb}	8.23±0.5^{Aa}		8.01±0.4^{Bb}
	30		8.11±0.3^{Aa}		7.89±0.3^{Ab}
	45		7.98±0.4^{Aab}		7.33±0.4^{bc}
	66		7.65±0.4^{Ab}		7.22±0.4^{Bc}
	87		7.03±0.4^{Ab}		7.02±0.2^{Ac}
	108		6.82±0.3^{Ab}		6.14±0.4^{Ad}
	142		6.18±0.4^{Abc}		
	180		6.01±0.3^{Ac}		
	228		6.00±0.2^{Ac}		
	Days	Control	HHP treated	Control	HHP treated
Flavor-aroma	0	7.03±1.4^{Aa}	8.46±0.4^{Aa}	7.00±0.4^{A}	8.22±0.4^{Aa}
	15	6.44±0.5^{Bb}	8.23±0.5^{Aa}		8.01±0.4^{Ab}
	30		8.11±0.3^{Aa}		7.89±0.4^{Ab}
	45		7.98±0.4^{Aab}		7.33±0.4^{Ac}
	66		7.65±0.4^{Ab}		7.22±0.4^{Ac}
	87		7.03±0.4^{Ab}		7.02±0.2^{Ac}
	108		6.82±0.3^{Ab}		6.14±0.3^{Ad}
	142		6.18±0.4^{Abc}		
	180		6.01±0.3^{Ac}		
	228		6.00±0.3^{Ac}		
	Days	Control	HHP treated	Control	HHP treated
Bitter taste	0	4.44±0.7^{Aa}	3.66±0.7^{Aa}	4.33±0.5^{A}	4.02±0.4^{Aa}
	15	6.41±0.6^{Cb}	3.67±0.4^{Aa}		4.08±0.3^{Bb}
	30		3.65±0.4^{Aa}		4.09±0.2^{Ab}
	45		3.76±0.3^{Aab}		4.03±0.2^{Bc}
	66		3.78±0.4^{Ab}		4.12±0.3^{Bc}
	87		3.80±0.3^{Ab}		4.22±0.3^{Ac}
	108		3.96±0.2^{Ab}		4.34±0.3^{Ad}
	142		3.98±0.4^{Abc}		
	180		4.04±0.3^{Ac}		
	228		4.02±0.3^{Ac}		
	Days	Control	HHP treated	Control	HHP treated
	0	4.42±0.5^{Aa}	3.44±0.4^{Aa}	4.33±0.5^{A}	4.02±0.4^{Aa}

Sour taste	15	6.98±0.6^{Cb}	3.40±0.4^{Aa}		4.08±0.3^{Bb}
	30		4.01±0.4^{Aa}		4.09±0.2^{Ab}
	45		4.02±0.4^{Aab}		4.03±0.2^{bc}
	66		3.78±0.4^{Ab}		4.12±0.3^{Bc}
	87		3.80±0.3^{Ab}		4.22±0.3^{Ac}
	108		3.96±0.2^{Ab}		4.34±0.3^{Ad}
	142		3.98±0.4^{Abc}		
	180		4.04±0.3^{Ac}		
	228		4.02±0.2^{Ac}		
	Days	Control	HHP treated	Control	HHP treated
After taste	0	6.78±0.2^{Aa}	7.84±0.4^{Aa}	6.32±0.6^{A}	7.04±0.4^{Aa}
	15	3.48±0.6^{Cb}	7.64±0.6^{Aa}		6.68±0.3^{Bb}
	30		7.33±0.4^{Aa}		6.29±0.4^{Ab}
	45		7.34±0.4^{Aa}		6.20±0.4^{bc}
	66		7.67±0.4^{Aa}		6.18±0.4^{Bb}
	87		7.56±0.4^{Aa}		6.08±0.4^{Bb}
	108		7.49±0.4^{Aa}		5.67±0.5^{Bb}
	142		7.41±0.4^{Aa}		
	180		7.38±0.5^{Aa}		
	228		7.26±0.5^{Aa}		

Data in the same column with different lowercase superscript letter and data in the same row with uppercase superscript letter are significantly different ($p \leq 0.05$)
